# Supplementary material for: Membrane-bound Interleukin-1α mediates leukocyte adhesion during atherogenesis
Source: Front Immunol. 2023 Aug 28;14:1252384. doi: 10.3389/fimmu.2023.1252384 (PMC10494239; doi:10.3389/fimmu.2023.1252384)

Supplementary Material

Membrane-bound Interleukin-1α mediates leukocyte adhesion during atherogenesis

Christina Maeder[1], Thimoteus Speer[2,3], Angela Wirth[4,5], Jes-Niels Boeckel[1], Sameen Fatima[5], Khurrum Shahzad[5], Marc Freichel[2,3], Ulrich Laufs[1] and Susanne Gaul[1]

[1] Klinik und Poliklinik für Kardiologie, Universitätsklinikum Leipzig, Leipzig University, Leipzig, Germany

[2] Klinik für Nephrologie, Universitätsklinikum Frankfurt am Main, Frankfurt am Main, Germany

[3]DZHK (German Centre for Cardiovascular Research), partner site Heidelberg/Mannheim, Germany

[4] Institute of Pharmacology, Heidelberg University, Heidelberg, Germany

[5] Department of Diagnostics, Laboratory Medicine, Clinical Chemistry and Molecular Diagnostic, Universitätsklinikum Leipzig, Leipzig University, Leipzig, Germany

***Corresponding author:**

Christina Maeder

Klinik und Poliklinik für Kardiologie,

Universitätsklinikum Leipzig,

Max-Bürger Forschungszentrum (MBFZ)

Johannisallee 30, 04103 Leipzig, Germany

Email: Christina.maeder@medizin.uni-leipzig.de

Supplements
Figure S1

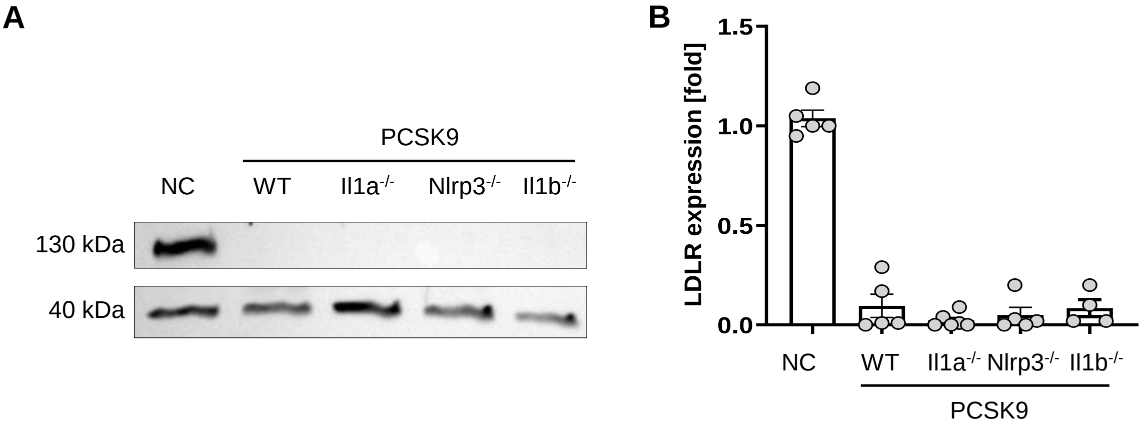

Supplements
Figure S2

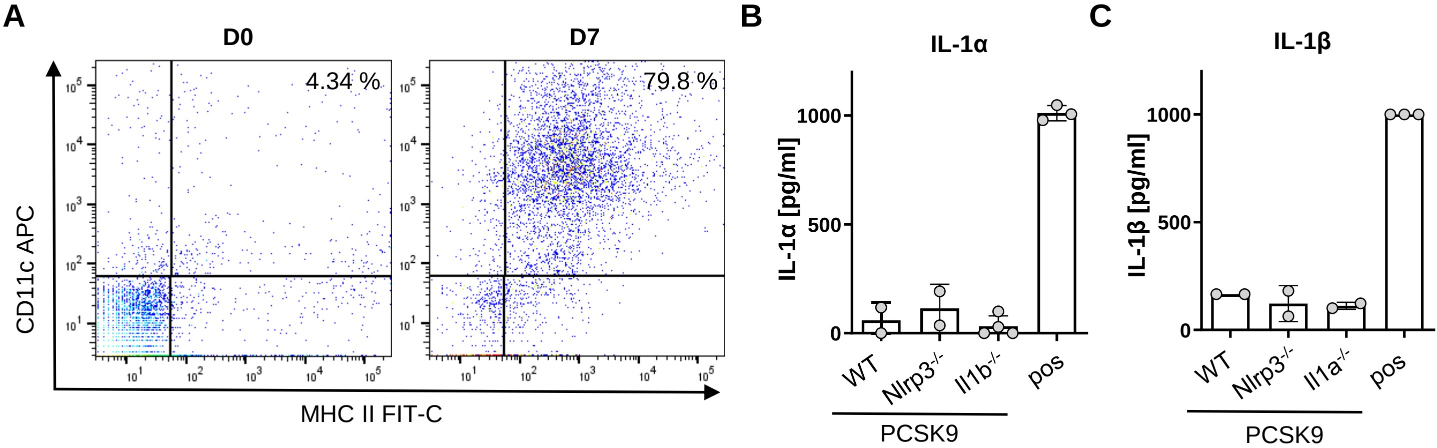

Supplements
Figure S3

A

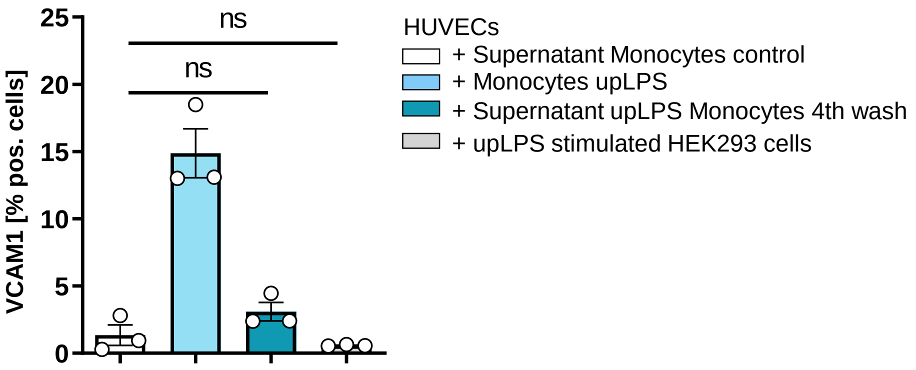

Supplement: Supplementary Figure 1 — A: Representative immunoblot of the LDL-receptor (LDLR) expression and β-actin expressed in liver tissue B: Densiometric quantification of LDLR normalized to β-actin. [file DataSheet_1.pdf]
